# Perspectives of healthcare providers, service users, and family members about mental illness stigma in primary care settings: A multi-site qualitative study of seven countries in Africa, Asia, and Europe

**DOI:** 10.1371/journal.pone.0258729

**Published:** 2021-10-27

**Authors:** Mirja Koschorke, Nathalie Oexle, Uta Ouali, Anish V. Cherian, Vayankarappadam Deepika, Gurucharan Bhaskar Mendon, Dristy Gurung, Lucie Kondratova, Matyas Muller, Mariangela Lanfredi, Antonio Lasalvia, Andrea Bodrogi, Anna Nyulászi, Mario Tomasini, Rabih El Chammay, Racha Abi Hana, Yosra Zgueb, Fethi Nacef, Eva Heim, Anaïs Aeschlimann, Sally Souraya, Maria Milenova, Nadja van Ginneken, Graham Thornicroft, Brandon A. Kohrt

**Affiliations:** 1 Centre for Global Mental Health, Institute of Psychiatry, Psychology and Neuroscience, King’s College London, London, United Kingdom; 2 Department for Psychiatry II, Ulm University and BKH Günzburg, Günzburg, Germany; 3 Department of Psychiatry A, Razi Hospital La Manouba, Manouba, Tunisia; 4 Faculty of Medicine of Tunis, University of Tunis El Manar, Tunis, Tunisia; 5 Department of Psychiatric Social Work, National Institute of Mental Health and Neurosciences, Bengaluru, India; 6 Department of Epidemiology, Centre for Public Health, National Institute of Mental Health and Neurosciences, Bengaluru, India; 7 Transcultural Psychosocial Organization Nepal, Kathmandu, Nepal; 8 Department of Public Mental Health, National Institute of Mental Health, Klecany, Czechia; 9 Unit of Psychiatry, IRCCS Istituto Centro San Giovanni di Dio Fatebenefratelli, Brescia, Italy; 10 Section of Psychiatry, Department of Neuroscience, Biomedicine and Movement Sciences, University of Verona, Verona, Italy; 11 Awakenings Foundation Budapest, Budapest, Hungary; 12 Department of Mental Health, Alto Garda e Ledro Giudicarie, Arco, Italy; 13 National Mental Health Programme Ministry of Public Health, Beirut, Lebanon; 14 Department of Psychiatry, Saint Joseph University, Beirut, Lebanon; 15 Department of Clinical, Neuro and Developmental Psychology, Vrije Universiteit, Amsterdam, The Netherlands; 16 Institute of Psychology, University of Lausanne, Lausanne, Switzerland; 17 Department of Psychology, University of Zurich, Zurich, Switzerland; 18 Implemental Worldwide, London, United Kingdom; 19 Centre for Implementation Science, Institute of Psychiatry, Psychology and Neuroscience, King’s College London, London, United Kingdom; 20 Institute of Population Health Sciences, University of Liverpool, Liverpool, United Kingdom; 21 Division of Global Mental Health, Department of Psychiatry, George Washington University, Washington, DC, United States of America; National Health Laboratory Service, SOUTH AFRICA

## Abstract

**Background:**

Stigma among healthcare providers is a barrier to the effective delivery of mental health services in primary care. Few studies have been conducted in primary care settings comparing the attitudes of healthcare providers and experiences of people with mental illness who are service users in those facilities. Such research is necessary across diverse global settings to characterize stigma and inform effective stigma reduction.

**Methods:**

Qualitative research was conducted on mental illness stigma in primary care settings in one low-income country (Nepal), two lower-middle income countries (India, Tunisia), one upper-middle-income country (Lebanon), and three high-income countries (Czech Republic, Hungary, Italy). Qualitative interviews were conducted with 248 participants: 64 primary care providers, 11 primary care facility managers, 111 people with mental illness, and 60 family members of people with mental illness. Data were analyzed using framework analysis.

**Results:**

Primary care providers endorsed some willingness to help persons with mental illness but reported not having appropriate training and supervision to deliver mental healthcare. They expressed that people with mental illness are aggressive and unpredictable. Some reported that mental illness is incurable, and mental healthcare is burdensome and leads to burnout. They preferred mental healthcare to be delivered by specialists. Service users did not report high levels of discrimination from primary care providers; however, they had limited expectations of support from primary care providers. Service users reported internalized stigma and discrimination from family and community members. Providers and service users reported unreliable psychiatric medication supply and lack of facilities for confidential consultations. Limitations of the study include conducting qualitative interviews in clinical settings and reliance on clinician-researchers in some sites to conduct interviews, which potentially biases respondents to present attitudes and experiences about primary care services in a positive manner.

**Conclusions:**

Primary care providers’ willingness to interact with people with mental illness and receive more training presents an opportunity to address stigmatizing beliefs and stereotypes. This study also raises important methodological questions about the most appropriate strategies to accurately understand attitudes and experiences of people with mental illness. Recommendations are provided for future qualitative research about stigma, such as qualitative interviewing by non-clinical personnel, involving non-clinical staff for recruitment of participants, conducting interviews in non-clinical settings, and partnering with people with mental illness to facilitate qualitative data collection and analysis.

## Introduction

Stigma against mental illness is “the main obstacle to the provision of care for people with mental disorders,” [1] p.810. The World Health Organization (WHO) called for stigma eradication as one of the central pillars in its Action Plan 2013–2020 [2]. In low- and-middle-income countries (LMIC), where the emphasis has been on primary care providers (PCPs) delivering mental healthcare [3], there is desperate need to eradicate stigma in primary care centers. Unfortunately, a global consensus package of guidelines and evidence-based interventions is lacking for stigma reduction in LMIC and low resource settings in high-income countries (HIC). The current evidence base has gaps in evaluation of behavior change, long-term outcomes, and multi-tiered system approaches that are needed for primary care settings [4–6].

As efforts for mental health training of PCPs are expanded in both LMIC and HIC, there is an opportunity to identify how delivery of care is affected by stigma and to reveal the experience of people with mental illness who receive their care in primary care centers. To date, rollouts of mental healthcare in primary care have reported limited uptake from trainings into service provision, combined with the lack of fidelity to evidence-based care among non-specialists, which are barriers to scaling up effective care [7]. This arises, in part, from stereotypes and negative attitudes among non-specialists, such as beliefs that people with mental illness are violent, have a contagious illness, are to blame for their illness, and can only be treated by specialists [4, 8, 9]. For scale-up of mental health services to succeed, there is a need to tackle the widespread intentional and unintentional discrimination against people with mental illness in primary care globally [9, 10]. Ultimately, a better understanding of stigma in primary care settings has the potential to address sustainability of attitudinal change, translation of attitudes into clinical competence, and the redesigning of health infrastructures to maintain positive attitudes [4, 11, 12].

Therefore, the aim of this study was to document perspectives of PCPs and people with mental illness who use primary care services. The results of this study will serve to inform the development of international tools and stigma reduction guidance for intervention guides, such as the WHO mental health Gap Action Programme (mhGAP) [13], and ongoing efforts of consortia tackling mental health stigma [14], thereby contributing to improving access to and effective care for those living with mental illness, and overall enhancement of their rights, quality of life, and social inclusion.

## Methods

### Study design

The study was carried out as a component of the International Study of Discrimination and Stigma Outcomes (INDIGO) global research network [14]. Within INDIGO, the “PRogramme for Interventions addressing Mental healthcare knowledge, Attitudes and behavior in primaRY care” (PRIMARY) initiative aimed to develop, implement and evaluate interventions to improve knowledge, attitudes and behavior of PCPs towards service users in primary care settings.

The current study is one of the initial steps in the INDIGO-PRIMARY initiative. This is a qualitative situational analysis carried out in diverse participating sites that aimed to understand experiences in primary care centers. The study sought to cover the perspectives of PCPs, service users, and their families, as well as other stakeholders in primary care settings in Asia, Africa and Europe. Partner investigators and institutions in the broader INDIGO research network were given the opportunity to volunteer to participate in this multi-country qualitative situational analysis. The participating sites included four LMIC in South Asia, the Middle East, and North Africa (India, Nepal, Lebanon and Tunisia) as well as three HIC in Europe (Czech Republic, Hungary, and Italy). There were no exclusion criteria for sites. The only requirement was that partners would be able to implement the qualitative research without funding, and that a common qualitative protocol would be used. The study was carried out without project-specific funding in all sites except India, where a local grant was obtained. The unfunded nature of the study meant that feasibility considerations determined the recruitment strategies and sample sizes in each site.

Methods comprised the collection and analysis of qualitative data arising from original interviews and focus groups with PCPs, service users, and their families as well as other stakeholders. The qualitative data included in the analyses comprised largely of information collected purposely for the INDIGO-PRIMARY study. In Nepal, the study team had recently collected relevant qualitative information on the study subject [15–19], so these data were used and analyzed afresh for the purposes of this study. In the Czech Republic, new INDIGO-PRIMARY data collection was supplemented with comparable qualitative data from other local studies. All site leads carried out a documentary analysis of health policy and health system documents relevant to mental health in primary care in their site (these findings will be published in detail separately).

Qualitative data were collected by local researchers in the local languages in each site. Participation in the study was initiated after providing written informed consent. Care was taken to carry out interviews in settings to maintain privacy and not hinder participants’ free expression. After audio recording, interviews were transcribed by the researchers who had conducted them and analyzed in the local language. The exceptions were in Tunisia and Lebanon, where interviews were translated into English and then analyzed by international interdisciplinary research teams. Full details for the Consolidated Criteria for Reporting Qualitative Studies (COREQ) are provided for each site in S1 Table.

### Participants

Participants for individual interviews and focus groups consisted of:

1. *Primary Care Providers (PCPs)*: frontline providers working in participating primary care centers who had received general training and were not mental health specialists. These included professional providers (doctors, nurses, midwives) whose primary responsibilities were patient care, non-professional providers (such as community health workers and lay providers), and administrative and support staff. In addition, supervisors for primary care facilities, such as primary care managers or lead primary care clinicians, were included.
2. *People with mental illness (service users) and family members*: persons receiving treatment for a mental illness at the primary care centers and their family members. Service users were recruited from the primary care centers based on nomination from PCPs about patients who received care there for mental illness. In addition, because of other ongoing research and clinical activities, study site teams were familiar with service users who obtained mental healthcare in primary care settings, and these individuals were also recruited. For the purposes of this study, we have referred to service users with *common mental disorders* as those experiencing depression, anxiety, and posttraumatic stress disorder. Service users with *severe mental disorders* refers to those with schizophrenia or other psychotic conditions, as well as bipolar affective disorder. We use this convention for classification because it was a common categorization used by respondents. We also acknowledge that depression, anxiety, and posttraumatic stress disorder can be accompanied by severe symptomatology and severe disability. The common vs. severe distinction is not an indication of the impact the disorder on an individual’s life and wellbeing.

Efforts were made to ensure that participants in the sample were reasonably representative of PCPs and service users with experiences in mental healthcare integrated into primary care in the different settings. Interviews were also collected with a few mental health professionals collaborating with the primary care facilities and policy makers or health authority representatives. However, not all countries participating in INDIGO-PRIMARY were able to complete this. Therefore, the current analysis will only focus on findings involving comparable respondents across all sites (i.e., PCPs, service users, and their family members).

In the Czech Republic, researchers had at least a master’s degree or higher and they were affiliated with the National Institute of Mental Health in the Czech Republic. Some of the researchers had clinical training. For PCPs, recruitment took place and interviews were conducted in their primary care centers. For PCPs, purposive sampling was used to obtain individuals with different professional roles and clinical backgrounds. For service users, the researchers approached them and interviewed them in clinics. Service users were approached when researchers were available to attend clinics, and thus it was a sample of convenience. No one approached for the study refused. All interviews were audio recorded.

In Hungary, inclusion criteria for PCPs were professional training or a degree in health care and active contact with patients. Inclusion criteria for service users were diagnosis of a mental illness and active involvement in psychiatric treatment. In Hungary, the interviewer orally informed the study participants about the study and provided them with an information sheet and obtained a written form of consent. As the resources allowed us to use a convenience sample, the participants were contacted through the Hungarian team’s lead researcher (a psychiatrist) in the district of the Community Psychiatry Centre. The healthcare professionals of the primary care centers were interviewed by a clinical psychologist trainee. Respondents’ answers were transcribed during the interview on paper. The interview duration was 45–90 minutes. The service users and the family members were interviewed by the site’s senior psychiatrist. The interview duration was 60–90 minutes and with notes transcribed on paper. No video or audio recording was utilized to mitigate participants’ concerns of confidentiality breaches. All PCPs, service users, and family members agreed to be interviewed, and none of the participants refused to take part in the study.

In India, the study sites selected were in rural geographic areas. The study team approached the District Mental Health Programme consultant (a psychiatrist) for coordination of the study at the site and the project lead and the consultant together supervised the activities of the researcher at the study site. With the help of the District Mental Health Programme team, three rural primary care centers were selected out of sixty primary care centers based on the demographic details, referral rates and cooperation for the study. The team also provides supervision of mental health services at primary care centers and also trains PCPs on mental health. At the primary care centers, the study sample was selected based on purposive sampling: the available PCPs who were willing to participate in the study were included. Service users were identified and approached for the interviews with the help of primary care staff. Service users who were symptomatic or cognitively impaired were excluded. Two service users and one PCP refused to participate in the study. However, there was no dropout of participants. The research staff who had research experience underwent special training in qualitative research by the project lead, who has previous experience in qualitative research. An interview guide prepared by the INDIGO team was translated to the local language and used for the interviews. In-depth interviews with participants were carried out at the selected primary care centers and each interview lasted for 60 to 90 minutes. The interviews were audio recorded and the verbatims were translated and transcribed. The transcripts were reviewed by an independent researcher. Subsequently, the research team carried out the qualitative data analysis, and the project lead and co-lead who were experts in qualitative research supervised and reviewed the data analysis process.

In Italy, PCPs were those potentially involved in the care of patients with a mental illness. In Italy, all primary care providers are the first-line professionals for people with illnesses, as they are the gatekeepers for specialized mental health services. For service users, the only criterion was that he/she had to receive care for a mental health problem, regardless of diagnosis. For caregivers, the only criterion was that he/she had to be a first-degree relative of a person with a mental illness, regardless of diagnosis. Initially, potential participants were contacted by the lead researcher via telephone or e-mail and asked if they were interested in participating. In the case of consent, participants were subsequently approached by the local research team for the interviews. Participants were first given full details on the aims and the overall design of the study; informed consent forms were signed by participants (with the exception of telephone interviews). Interviews lasted on average 30–60 minutes. Interviews with PCPs, service users, and family members were conducted face-to-face. Only the interview with the primary care manager was made on the telephone.

In Lebanon, primary care centers providing mental health services were included and PCPs trained on mhGAP were recruited for interviews. The primary care department of the Ministry of Population and Health contacted the directors of the primary care centers. The director decided whether to accept for his/her staff to participate in the study. Service users receiving care at the primary care centers were invited to participate. Only service users in psychiatric emergencies were excluded from the option to participate. Participants were contacted by primary care clinic focal point employees who were responsible for explaining the study and obtaining verbal agreement from participants. A member of the local research team orally presented the study to participants and provided them with a participant information sheet to peruse. Except when the interview was performed over the phone, all participants then completed and signed an informed consent form. When the phone conversation was set, the interviewer repeated the explanation and the consent form to the participant. The interviewer ensured that the participant had enough time to ask questions in advance. Phone interviews were conducted for service users who preferred it, and consent was obtained verbally in these circumstances. The interview duration was 30 to 60 minutes. Although all staff agreed to be interviewed, some service users did not feel comfortable doing the interview because the first contact was done by primary care clinic focal persons. However, the exact number of service users who refused to participate was not recorded.

In Nepal, existing qualitative datasets with PCPs, service users and their family members, and policymakers [15–19] were used to extract information relevant to the INDIGO-PRIMARY topics of interest. Inclusion criteria for PCPs were those who received mhGAP training through the PRogramme for Improving Mental healthcarE (PRIME) project in Nepal [20] and who consented to participate in all research activities including training evaluations and qualitative interviews in the beginning of the training. Inclusion criteria for service users were: being identified as having one of the four priority disorders of the project (depression, alcohol use disorder, epilepsy, or psychosis), being above the age of 18 years, and having received treatment from the PCPs who received the mhGAP training. All the interviews were conducted by trained research assistants in the Chitwan district of Nepal, where the projects were being implemented. The participants were selected by the study teams in coordination with the PCPs from local primary care centers. In-depth interviews were conducted with PCPs, service users, and family members in all the projects. Written consent was sought from the participants prior to the interview. The interviewers followed a semi-structured interview guide to conduct the interviews. For the service users and family members receiving treatment in primary care centers, the questions focused on their explanatory models, help-seeking, barriers and facilitators to their treatment, and reasons for non-adherence (if they had dropped out of treatment). For PCPs, questions focused on their experience of treating mental health patients (barriers and facilitators), receiving mhGAP training, knowledge, and attitude. Similarly, for service users involved in stigma interventions [15, 21, 22], questions focused around their experiences of participating in mhGAP trainings with PCPs. All interviews were conducted in Nepali and were audio-recorded. They were then transcribed and translated by professionals.

In Tunisia, the site was chosen based on acceptance of the lead PCP to conduct the study in her primary care center, and based on placement of the primary care center in the geographical sector of the referent psychiatry department. All service users of this particular primary care center were approached to participate in the study, and two refused. In Tunisia, senior psychiatrists, who were clinician-researchers at the local hospital, conducted the qualitative interviews. They had qualitative research training, with prior experience leading key informant interviews and focus group discussions. The psychiatrists conducting the qualitative interviews was known to some of the participants including the lead PCP, the associated mental health professional, and the program manager participating in the study. But the psychiatrist conducting interviews was not known to the remainder of the study participants.

### Qualitative situational analysis tool

The situational analysis tool comprised questionnaires for documentary analysis as well as topic guides for qualitative interviews and focus group discussions. The questionnaires for the documentary analysis were based on an existing case study methodology outlined for mental health projects in LMIC [23] as well as a situational analysis tool developed for a large international program of mental health interventions in primary care [24]. The topic guides were adapted from guides used for the exploration of experiences of stigma and discrimination among people with mental illness in India [25], which have also been used in other studies on stigma and discrimination in India [26].

As INDIGO-PRIMARY was carried out in culturally and socio-economically diverse sites, core topic guides comprised a list of broad questions and probes with the phrasing of questions developed locally to take the cultural and contextual factors of each site into account. The situational analysis tool is accessible online (see S1 Table).

### Data analysis

Techniques of framework analysis were used for qualitative data, allowing for analysis both by site and across sites. This approach was chosen as it compares cases and codes, making analysis and dissemination digestible and relevant to policy-makers [27]. A thematic coding framework was created jointly with the research teams in all sites to structure multiple-researcher coding [28]. Inductive coding was used to identify additional codes relevant to the specific context in each site. The coding framework was charted into tables to compare various stakeholders’ experiences and views on key themes such as “attitudes of PCPs” or “experiences of stigma and discrimination in primary care” [27]. In a second step, similarities and differences between research sites were mapped out on each theme. Relevant subthemes evident in the data were identified (e.g., different types of stereotypes evident in PCP narratives across countries). Cross-country findings on each theme were summarized for a) data from PCPs (provider perspective) and b) data from service users/family members (service user perspective), with a particular focus on findings that were salient across study sites and findings that appeared to be more locally specific. In a final step, implications for interventions to address stigma and discrimination in primary care were identified.

Regarding presentation of results, we have chosen to use non-specific terminology rather than to present results as percentages or frequencies. We are aware that this issue is debated among qualitative researchers. We are following one of the recommended conventions in presenting qualitative research [29] that we feel best fits the nature of our study design and data. Neale and colleagues (2014) recommend that raw numbers be only included for features that have been assessed for all participants. Because of the semi-structured nature of these interviews, we cannot state with certainty that a particular subtheme as presented in our results was presented in manner that allowed every participant to respond with an endorsement or denial of the statement. This is better suited to a structured questionnaire, which is something that could follow from this research to establish prevalence of different attitudes or experiences in a multi-site study. In addition, Neale and colleagues recommend that reporting % be limited to studies in which the sample size is at least 50 respondents are greater. Although our total sample is 248, all sites except Nepal are below the 50-respondent threshold. Therefore, any reporting of percentages would be biased toward the Nepal results and not representative across countries. Neale and colleagues discourage using terms with specific quantitative meaning (e.g., majority, minority) with a specific justification, and instead recommend non-specific language (e.g., few, several, some, many). Ultimately, we chose this latter strategy of using non-specific terms when helpful to convey meanings, and we have avoided use of percentages, frequencies, and specific quantitative terminology. This best captures the spirit that qualitative data should be used to understand phenomena rather than quantifying frequency of themes to infer prevalence of such phenomena [29].

### Ethical statement

Informed consent was obtained from all participants taking part in data collection for this study. Ethics approval was obtained from the PNM Research Ethics Subcommittee, King’s College London (Reference Number (No.) RESCMR-17/18-4109; approval date 23 February 2017) and local Ethics Committees in the participating sites: Czech Republic (National Institute of Mental Health, Klecany, Czechia. No 128/17, 19.4.2017), Hungary (Semmelweis Egyetem Regionális, Intézményi Tudományos és Kutatásetikai Bizottság, No. SE TUKEB 162/2017), India (NIMHANS Institutional Review Board, Ref No: NIMHANS/IEC(BEH.SC.DIV.)7th MEETING/2017; and permission from Directorate of Health Family Welfare Servicers, Government of Karnataka, India, No: DD/Mental Health/10/18-19 dated 26.04.2018), Italy (Comitato Etico IRCCS San Giovanni di Dio–Fatebenefratelli, Brescia, No. 102/2017; Ethics Committee of the Provinces of Verona and Rovigo, No. 44066, 13 September 2017), Lebanon (Saint Joseph’s University Beirut, No. CEHDF 1193; 3 July 2018), Nepal (Nepal Health Research Council, No. 139/2016, 28 July 2016), Tunisia (Ethics Committee of Razi Hospital La Manouba, 28 January 2017).

## Results

### Description of settings

The studies were conducted in primary care settings in seven countries, with all countries having different models of primary care-based mental health services (see Tables 1 and 2). We first describe the context in the LMICs: In India, three primary healthcare centers in the Ramanagaram District, Bengaluru Division, of Karnataka State in the southern central region of the country participated in the qualitative situational analysis. In Bengaluru, there is a history of initiatives to support primary care-based mental health services going back multiple decades [30]. In Nepal, there has been a recent initiative through PRIME, through which a district mental health plan was developed that included training primary care workers on mhGAP and psychosocial skills [20]. The PRIME initiative was conducted in southern Nepal in the Chitwan district bordering India. The prior qualitative study from which data were drawn for this study included 32 primary care centers for anti-stigma study conducted in those settings [15]. In Lebanon, two primary care centers participated in the Mount Lebanon area. Some of the participating Lebanese PCPs were are also trained on mhGAP, and community mental health centers provide specialized mental health services. However, not all PCPs had mhGAP training at the facilities. This represents the situation throughout the country where only some PCPs have had exposure to mental health training. In one primary care center in the Greater Tunis area of Tunisia, some providers have been exposed to training through the national mental health program, although none had received formal mhGAP-IG training. Among those who have had training, PCPs generally have limited responsibilities in mental healthcare with the exception of basic medication management for depression and anxiety. In summary, the PCPs in the LMIC are representative from the perspective of a mix of those who have had and have not had a focused mental health training, and all recruited PCPs worked at primary care centers where some mental healthcare was being provided.

**Table 1:** Overview of mental health and primary care in participating countries: Low- and middle-income countries (LMICs).

	India	Nepal	Lebanon	Tunisia
**World Health Organization Region, World Bank Income Level**	South Asia, Lower-middle-income country	South Asia, Low-income country (at the time of the study)	Middle-East/North Africa, Upper-middle income country	Middle-East/North Africa, Lower-middle income country
**Mental Health Care System**	Mental health services are provided at primary, secondary and tertiary care settings, which includes both government and private practitioners. In the study site, mental health services are available at the district hospital and through the district mental health program, which consists of a multidisciplinary team. Mental health professionals are associated with all PCCs in the entire district.	Mental health services are currently available in few districts where community mental health services are introduced by both government and NGOs. PCCs who received mental health training from an NGO do provide some services; most mental health services are provided within tertiary hospitals, district level hospitals or private hospitals.	Mental health services are provided at some PCCs as part of the Ministry of Public Health network. Community mental health centers are also part of the National Mental Health Plan. Specialized clinics are available in the private sector. Eight private hospitals have psychiatric wards and one psychiatric ward was recently opened in a public hospital. The private sector is predominant.	Mental health services are provided by the public and the private sector. All inpatient facilities are in the public sector, most often at the 3^rd^ level of care. Outpatient services are provided by outpatient departments of a major psychiatric hospital in Tunis, by general hospitals and by private practitioners (psychiatrists).
**Pathways to Mental Healthcare**	The majority of patients seek help within the healthcare system (e.g., consult either a psychiatrist general practitioner); however, between 33–90% of patients (persons with psychotic disorders) consult faith healers and native healers before seeking help within the healthcare system.	Traditional and religious healers are known to be the primary sources of treatment for mental health problems in the community. It is estimated that over 75% of all illnesses are treated within the traditional healthcare system. Scholars found that home remedies for illness are often sought first, followed by formal health-seeking within the ‘traditional’ system.	Service users usually access treatment from specialized mental health clinics by consulting psychotherapists or psychiatrists, psychiatric hospitals, general hospitals with psychiatric wards and neurologists, but also traditional healers and religious leaders. Less persons visit primary healthcare centers for mental health services and in general people are used to going to specialists.	For depression and anxiety, people predominantly seek help in general medicine first (private and public family doctors), whereas for severe mental illness, an estimated 40% see a psychiatrist or are admitted to psychiatric hospital directly. Traditional or religious healers are still a primary source of treatment for all kinds of mental health problems.
**Primary Care System**	In India, PCPs exist in rural villages and are an integral part of the governmental healthcare system. Each PCC provides services for 20,000 persons (hilly and tribal areas) or 30,000 persons (plain areas).	PCPs in Nepal are health assistants, auxiliary health workers and medical officers delivering services through primary health centers and health posts established in each electoral area as a first referral point. Health posts and community health units are the lowest level facilities functioning in the community.	PCPs in Lebanon provide basic physical and mental health services (e.g., basic medication and health awareness). In addition, community mental health centers exist, which provide more specialized mental health services with a mental health multidisciplinary team. While there are numerous PCCs in urban areas, the number of PCCs in rural areas is low.	A wide network of PCPs exists within Tunisia which provide proximity care for the whole population (90% of population lives <5km from a PCC).
**Mental health in Primary Healthcare**	Mental health services at PCCs include identification of service users, condition diagnoses, medication provision, non-specific counselling, informal community outreach programs and referral services. ASHA workers and field health workers usually conduct home visits and provide necessary services.	The government is gradually rolling out an abbreviated version of WHO’s mhGAP 2.0 and basic psychosocial care module for primary health workers. The health workers can identify mental health problems and provide medical treatment as well as basic counseling or psychosocial supports.	Mental health services at PCPs include the identification of service users, basic management, and referral to specialized care when necessary. Community mental health centers are more specialized in providing mental health services (e.g., psychopharmacological medications, psychotherapy, and case management).	PCPs have a limited role in mental healthcare despite a national mental health program aiming to implement mental healthcare into primary care. For the time being, PCPs mainly provide psychotropic medication. The management of common mental disorders (mainly depression and some anxiety disorders) and follow up of severe mental illness is limited to some PCCs, where primary care clinicians are motivated and have received training.
**Primary Care Facilities Included in this Study**	Three primary healthcare centers in the Ramanagaram District, Bengaluru Division, Karnataka State.	32 primary care facilities in Chitwan district.	Two primary healthcare centers in Mount Lebanon.	One primary healthcare center in the Greater Tunis area.
	mhGAP training: No	mhGAP training: Yes	mhGAP training: Partial (one PCC had training, and some providers at the other facility had training)	mhGAP training: No
	Other primary care-based mental health training: Yes, Department of Mental Health Program	Other primary care-based mental health training: No	Other primary care-based mental health training: No	Other primary care-based mental health training: Some providers trained through National Mental Health Plan

Abbreviations: mhGAP, mental health Gap Action Program; PCC, primary care center; PCP, primary care provider.

**Table 2:** Overview mental health and primary care in participating countries: High-income countries (HICs).

	Czech Republic	Hungary	Italy
**World Health Organization Region, World Bank Income Level**	Europe, High-income country	Europe, High-income country	Europe, High-income country
**Mental Healthcare System**	Inpatient mental health services are predominantly concentrated in psychiatric hospitals [31]. Outpatient psychiatric care is provided via private profit and not-for-profit psychiatrists. Community mental health services are provided via NGOs but are not extensively developed in all regions. Ongoing mental health reform is developing community mental health centers which combine health and social care.	Mental health services are provided by both outpatient and inpatient mental healthcare units. In addition, there are not-for-profit mental health centers which provide treatment to people according to their residence (similar to PCCs).	Mental health services are provided by community mental health centers (mainly for people with severe mental disorders), general hospital psychiatric units (acute treatments), semi-residential facilities (day centers and day hospitals) and residential facilities offering therapeutic and rehabilitative programs.
**Pathways to Mental Healthcare**	Those seeking help for psychological or emotional problems usually turn to general practitioners (73%), less often to psychiatrists (7%) or psychologists (7%) [32]. Because there is not a developed system of early detection and early intervention services, a considerable number of patients at the first stage of a severe mental illness get directly admitted to psychiatric hospitals.	Generally, family doctors in PCCs refer persons with mental illness to mental health centers, where they get outpatient treatment. If any patients need inpatient treatment, specialists in outpatient mental health centers refer them to hospitals with inpatient psychiatric departments.	Pathways to mental healthcare in Italy are characterized by a high proportion of patients reaching psychiatric services through direct access (34%) and with short delay, whereas the others arrive to specialist mental health services through general practitioners (33%), general hospitals (20%), or private practitioners (9.8%). Most patients with severe mental disorders are treated within mental health services, whereas GPs usually tend to treat patients with insomnia and anxiety/somatization disorders.
**Primary Care System**	In most cases, primary care is provided at *policlinics*, i.e., facilities that mostly provide outpatient care and comprise medical services of various specialties for less severe conditions. Primary care may be also provided within an outpatient clinic or hospital. General practitioners provide referrals to specialized services and are paid by insurance companies based on the number of registered patients.	In Hungary, there is a vast number of PCCs, all paid by the insurance company according to the number of registered patients. There are no private primary care centers.	Primary healthcare must cover all areas (rural and urban), and their distribution is proportional to the density of the population. PCCs are groups of single freelance general practitioners who work in an integrated way with nurses, administrative staff, social workers and medical specialists. General practitioners do not act as gatekeepers to secondary care, as there is open access to all levels of care (primary, secondary and tertiary care).
**Mental Health in Primary Care**	The majority of psychological support for common mental disorders is provided in primary care [32]. Mental health services are provided in primary care, including referrals to mental health professionals as well as prescription of antidepressants. General practitioners go through courses of psychiatry during their medical training but once they choose their specialization, there is no further systematic training in mental healthcare.	None of the PCCs provide mental healthcare. The family doctors send patients with mental illness to mental healthcare services, where they get treatment. With a written permission of a specialist mental health doctor, the family doctor has the right to prescribe antidepressant and anxiolytic medication to service users for a certain period (half year or one year).	PCPs identify mental illness and refer patients to specialized services when necessary. PCPs also treat common psychiatric disorders (e.g., anxiety disorders, mood disorders). Serious psychotic disorders are mostly managed by mental health departments. PCC doctors are authorized to prescribe and/or to continue prescription of psychopharmacological medication.
**Primary Care Facilities Included in this Study**	Three primary care facilities in Prague.	Four primary care facilities in Budapest.	Two primary care facilities in Brescia and one in Verona
	mhGAP training: No	mhGAP training: No	mhGAP training: No
	Other primary care-based mental health training: Yes, government program	Other primary care-based mental health training: Yes, government program	Other primary care-based mental health training: Yes, government program

Abbreviations: mhGAP, mental health Gap Action Program; PCC, primary care center; PCP, primary care provider.

Regarding the HICs: in the Czech Republic, three primary care centers in Prague participated in the study. Czech general practitioners receive general psychiatry training in medical school, but there is no further systematic mental health training for these doctors working in primary care. Their mental healthcare activities are limited to prescribing antidepressants and referring patients to mental health specialists. In Hungary, four primary care centers in Budapest participated in the study. Family doctors in primary care centers are responsible for referring patients to mental health specialists. Then with written permission from a specialist, family doctors can prescribe antidepressants and anxiolytics for a limited time of 6 months to 1 year. In Italy, two primary care centers in Brescia and one primary care center in Verona participated in the study. Italy has a long tradition of primary care- and community-based mental health services. PCPs detect mental illness and refer patients to specialists. PCPs are authorized to prescribe psychiatric medication, and they primarily treat common mental disorders.

To classify the types of primary care models of mental healthcare integration, we draw upon the framework adapted by van Ginneken and colleagues [30]. This framework classifies primary care mental health services into five types: 1) education and training models where PCPs are trained to independently manage mental illness; 2) consultation-liaison models in which PCPs independently manage with only educational support from specialists; 3) collaborative care models where care managers are in place to foster linkages among primary care centers and specialists; 4) referral/replacement models in which PCPs identify and refer patients to specialists; and 5) specialist-community models in which PCPs are trained within specialty programs to treat severe mental disorders in community settings. Models in all the LMIC partner sites (India, Nepal, Lebanon, and Tunisia) can be described as generally consultation-liaison. In the Czech Republic and Hungary, the model is predominantly replacement/referral. In Italy, the model is predominantly collaborative care.

### Sample description

Qualitative data included 248 total participants: 64 primary care providers, 11 primary care facility managers, 111 people with mental illness, and 62 family members of people with mental illness (see Table 3). Across the sites, the majority of primary care providers were women (43 women vs. 18 men), with India, Lebanon, and Tunisia each only having one male PCP. In the other sites, only 2–3 PCPs were men. PCPs were roughly equally divided among doctors and nurses. In some sites, e.g., Nepal, PCPs were managed by health auxiliary staff who did not have a medical degree (e.g., neither MBBS nor MD credentialing). Across sites, the number of participating PCPs was equally divided between those who did vs. did not receiving mental health training. In India, Nepal, and Lebanon, the majority had prior training. For example, in Lebanon and Nepal, the primary care doctors and primary care workers, respectively, had received mhGAP trainings. In Tunisia, the Czech Republic, and Hungary, the majority did not have prior mental health training. The majority of service users for whom gender information was available were women (21 women vs. 6 men). In the study, information on the diagnoses of service users was not available for nearly half of the participants; the remainder were comparably divided between common and severe mental illness. For those service users where age was recorded, most were older: 22 service users (90%) were 40 years of age or above compared to two under 40 years of age. Family members were comparably divided between men and women, with all family members 40 years of age or older.

**Table 3:** Qualitative sample participant demographics.

Stakeholder Group	India	Nepal^a^	Lebanon	Tunisia	Czech Republic^b^	Hungary	Italy	Total
***Primary Care Providers (PCPs)***								
Men	1	10	1	1	2	0	3	**18**
Women	14	10	5	9	3	3	2	**43**
Doctor	3		2		4		3	**12**
Nurse/ Pharmacist	5	10	2	7	1	3		**27**
Medical Auxiliary Worker	4	10						**14**
Receptionist/Admin Staff			1	2			2	**5**
Community Health Worker/ Midwife	3			1				**4**
Social Worker			1					**1**
Age 19 to 39	8	0		5				**13**
Age 40 or above	5	0		5	5	3		**18**
Age not known	2	20	6				5	**33**
No prior mental health training	3		2	8	3	3	5	**24**
Any prior mental health training	12	20	4	2	2	0		**38**
Not known		20						**20**
**Subgroup total**	**15**	**20**	**6**	**10**	**5**	**3**	**5**	**64**
***Lead primary care clinicians/ managers***								
Men			1		1	2	2	**5**
Women			1	3		2		**6**
**Group total**	**0**	**0**	**2**	**3**	**0**	**4**	**2**	**11**
***Service Users***								
Men	3			3				**6**
Women	6		2	5	1	7	1	**21**
Gender not recorded					28			**28**
Common mental disorder	5			3		5	1	**14**
Severe mental disorder	2			5	1	2		**9**
Diagnosis not known	2	55	2		28			**87**
Age 19 to 39	1					1		**2**
Age 40 or above	7			8	1	6	1	**22**
Age not known	1	55	2		28			**86**
**Subgroup total**	**9**	**55**	**2**	**8**	**29**	**7**	**1**	**111**
***Family members***								
Men	3				1		1	**4**
Women	2			3		2		**7**
Age 19 to 39								**0**
Age 40 or above	5			3	1	2	1	**11**
Age not known		50						**50**
**Subgroup total**	**5**	**50**	**0**	**3**	**1**	**2**	**1**	**62**
**Total**	**29**	**125**	**10**	**24**	**35**	**16**	**9**	**248**

^a^ In Nepal, data recently collected for a range of similar studies was included in the analysis. Primary care provider data are taken from [15]; people with mental illness and family members from [16, 17]; and primary care managers, mental health professionals, and policy makers from [18].

^b^ In Czech Republic, data (4 focus groups with a total of 28 people with mental illness, and 2 male general practitioners) collected for one similar project (Destigmatization of people with mental illness in Czechia) was included in the analysis, in addition to the data collection for this study (INDIGO-PRIMARY). For people with mental illness, details regarding their gender, age and diagnosis were not available.

There was considerable heterogeneity within and between study sites in the types and backgrounds of respondents. In Hungary, the study examined the work of PCPs in four primary care centers. The PCPs included four family physicians and three primary care nurses working in Budapest. The doctors in all four primary care centers are certified family physicians, having completed a specialist degree required in Hungary, and all four were lead clinicians. The medical primary care nurses all have completed education in nursing. In addition, all 4 clinics employ a receptionist, who has a high school degree. Two of the interviewed family physicians completed psychotherapeutic training, which is not compulsory for the training as general practitioner in Hungary. In India, there was a wide range of service providers from three different primary care centers, including medical officers, nurses, junior health workers, community health workers (Asha’s). Each professional category had the required qualifications as mandated by the government for their positions. For instance, medical officers were MBBS graduates, nurses had undergraduate nursing qualification, junior health workers had diploma degree qualifications, and Asha workers had the equivalent of a 10th grade education. The service providers interviewed had received very minimal training about mental health. In Nepal, the healthcare workers were ‘prescribers’ (i.e., medical officers with MBBS degrees, health assistants, and auxiliary health workers) who had prescription right as per the Government of Nepal and ‘non-prescribers’ (i.e., staff nurses and auxiliary nurse midwives) who did not have prescription rights.

### Primary care providers’ perspectives

#### Attitudes and behaviors of primary care providers toward persons with mental illness

Data from all study sites suggested that PCPs had interest in supporting service users and their needs, with the intention to treat them fairly. For example, PCPs actively tried to address the needs of service users: “*The way we treat the patient is what makes him feel at ease*. *We avoid raising our voice*. *We greet the patient*, *smile at him*, *and lead him to a private treatment room*,” *(Nurse*, *woman*, *Lebanon)*. Some PCPs even specifically tried to counter self-stigmatizing beliefs among service users with mental illness. However, many PCPs felt inadequately trained to deal with service users, and some expressed stigmatizing views and attitudes or reported behavior, which could have been experienced as devaluing or discriminatory by service users.

In all sites, there was evidence of stereotypes and misconceptions about mental illness and service users being held by PCPs: “*Stigma against people with mental health problems is present in society and in primary care workers alike*,” *(Lead PCP*, *woman*, *Tunisia)*. She added that this had improved over the years, thanks to training and contact with service users, so that fears of interacting with them had reduced but that there was still “*the idea that a mental patient is anyway somewhat crazy*, *capable of doing anything*.” Of note, the respondent did not distinguish if this response was limited to severe mental disorders, or if it also included common mental disorders, or if there was a local distinction.

The most consistent stereotype expressed by PCPs was that service users were aggressive, violent, dangerous, and lacked self-control. There were PCP statements from most sites endorsing these views. In India, PCPs held the view that people presenting with angry outbursts in the community probably had a mental illness. In Hungary, one female nurse described service users as much less patient and more aggressive than other physical healthcare service users. Similarly, a Tunisian PCP stated, “*Psychiatry for me is about people who hit you—offenders*, *borderline—who can hit you and are not accountable for it*,” *(PCP*, *woman*, *Tunisia)*.

The definition of what comprises a mental illness, ability to recognize these conditions, and the lack of effective treatments comprised another domain in which participants reported stigmatizing beliefs in some cases. Many PCPs either downplayed mental illness or deemed it incurable. In India, some PCPs reported that there were no cases of mental illness in their catchment area. In Hungary, one male doctor said that he considered alcoholism not to be a mental illness. In Italy, one doctor did not consider anxiety and depression psychiatric diagnoses, as they were so common. These perspectives reflected a range of issues such as lack of training in recognizing mental illness, lack of knowledge about effective treatments, and lack of exploration about topics such as when is general stress distinguishable from common mental disorders.

In India and Tunisia, some PCPs reported beliefs that mental illness was hereditary: “*During the training*, *I was informed about the chances of having mentally retarded children in case of marriage between people who have a blood relationship*, *about symptoms of mental illness*,” *(PCP*, *woman*, *India)*. In Tunisia, mental illness was commonly described as running within families with expressions such as “*the family has craziness” (Nurse*, *woman*, *Tunisia)*.

In India, Nepal, and Tunisia, some PCPs thought that mental illness was not curable and that pharmacological treatment was ineffective. Some doctors from India even stated that there was no use providing treatment for service users. “*No one recovers*. *I never saw anyone cured*. *Cured means that the treatment is finished*, *but in this case*, *it is a lifelong treatment*. *I have never seen anyone starting a psychotropic treatment and then stopping it*,” *(Nurse*, *woman*, *Tunisia)*. Similarly, another respondent explained, “*Medical treatment makes them ‘stumble against a wall’*,” *(Nurse*, *woman*, *India)*. In India, some PCPs commented that drug treatment could cure a patient if religious rituals also had been followed. A nurse reported that nurses and paramedics expressed helplessness when faced with service users: “*Lately there have been many cases (*…*) [the lead nurses] don’t know what to do about it*, *they say ‘they are weird’*, *(Nurse*, *woman*, *Czech Republic)*. These responses suggest interpretation of a medical model of infectious diseases, which are acutely treatable, versus ongoing chronic treatment needs, as well as beliefs particular to patients with mental illness suggesting low adherence with chronic treatment regimens.

Fear was a frequently stated emotion when PCPs talked about interacting with service users, especially when dealing with service users with challenging behaviors. This was expressed in all sites apart from Hungary. “*In the beginning*, *I was scared of them*, *as we have the idea that a … patient ill with his nerves may do anything…” (Nurse*, *woman*, *Tunisia)*. In India, some PCPs described men with mental illness as being "rough and tough" in nature, and therefore PCPs were scared to deal with them. Avoidance of service users was described in all LMIC settings. It was attributed to fear of patients and to the fact that staff did not know how to deal with them. As a consequence, PCPs stated that the best option was to refer patients with possible mental illness to specialist services. PCPs across all sites also expressed annoyance at the disruptiveness and time burden when engaging with service users. “*I think when you don’t know much*, *then there is this patient who is taking up a lot of your time and then there are 70 more to be seen*, *so obviously you’ll be irritated*,” *(PCP*, *man*, *Nepal)*.

PCPs in India, Nepal, Czech Republic, Lebanon and Tunisia shared examples of stigmatizing labelling they witnessed in their community for service users. They also reported that they themselves or their colleagues in the primary care center used these terms. In India, PCPs used terms such as “psycho”, whereas in Nepal, labels such as “*paagal* (crazy)” or “*baulaahaa* (mad)” were applied to describe service users. In the Czech Republic, PCPs used the word “schizophrenics” when referring to people with psychosis.

In Tunisia and the Czech Republic, PCPs also exhibited potentially paternalizing behaviors. They suggested service users should be treated in a more ‘careful’ way to avoid aggression or confrontation or that they should line up in a separate row to get medication more quickly to avoid problems: “*I think to the contrary [*we don’t treat people with mental illness worse than other people], *I think we treat them more carefully*. *We talk to them so that we prevent confrontation and aggression*,” *(Nurse*, *woman*, *Czech Republic)*.

*Structural factors and practices contributing to stigma in primary care*. Health providers of all sites apart from Italy described stigma as one of the main barriers to accessing care in any type of healthcare setting, including primary care centers. In some countries, PCPs reported that patients with mental illness were identified and labelled through the use of mental health cards (India) or visibly different patient files (Tunisia). In Tunisia, PCPs mark an “N” on health forms for patients receiving psychiatric medication. The lack of prioritization of psychiatric medication supply was also considered a devaluing of mental health services in primary care centers. Poor medication availability was a salient complaint in Tunisia and Nepal. Service providers of all sites reported lack of time allocated to sufficiently care for service users as leading to unreasonable workload and staff burnout.

*Expectations for mental health services in specialist facilities versus primary care*. PCPs reported a limited role in mental healthcare in the study settings in both HICs and LMICs. Clinicians and health workers at the participating PCPs identified mental illness and, in some cases, provided mental health treatments. However, they generally referred people with more severe mental illness to secondary or tertiary care, both in HIC and LMIC. In Italy and Hungary, some mental healthcare centers were available at the community level, and the same is planned for the Czech Republic. In Hungary, on the other hand, three primary care doctors stressed their central role in treating people with mental disorders. Where mental health treatments were offered in primary care, these predominantly comprised of pharmacotherapy. However, in some HIC settings and in Lebanon, psychotherapy was sometimes provided.

Many PCPs in Tunisia, India, and Nepal and several from Lebanon often did not see it as their role to provide mental health treatments. For example, one female nurse in Tunisia said that “*For the patients*, *seeing the psychiatrist is better than seeing us*. *It is true*, *they feel better*. *We do not replace the psychiatrist; we need to admit it*. *Why lie to ourselves*?*” (Nurse*, *woman*, *Tunisia)*. A doctor in Lebanon stated that “*Yes I am comfortable for providing psychiatric treatment*, *such as talk therapy*, *but it’s not my job*,” *(PCP*, *man*, *Lebanon)*. In Lebanon, a few community centers have been developed in collaboration with the National Mental Health Program and other partners; additional specialized mental health services are provided by NGOs, and collaboration is ongoing to transform these into community mental health centers. However, there is limited coordination with primary care centers.

In India, a doctor reported that delivering mental health services in primary care did not ameliorate the stigma; patients were also ashamed to get this care in primary care centers: “*If patients come for taking treatment*, *they close doors*, *express their problems as a secret*, *get drugs by making sure that their neighbors are not present near to them*. *People have fear of labelling and we also do not disclose any information about them to others*,” *(PCP*, *woman*, *India)*.

*Clinical competence and training experiences and needs*. In the majority of sites, PCPs reported a lack of knowledge about mental health problems and psychiatric medication. They specifically described difficulties in managing challenging situations related to agitation or other behavioral challenges as well as in managing patients who have drug problems. If at all, only people with common mental disorders were treated in primary care, whereas patients with severe mental illness were referred to specialist psychiatric services. There were some exceptions to this, such as in Nepal, where the mhGAP training curriculum had also included training on recognition and treatment of psychosis.

PCPs had differing views on their role in providing mental health services at the primary care level. Some PCPs in Tunisia and Lebanon felt that treating a person with mental health problems was not their role but the role of specialists. In Nepal, where mhGAP training had been conducted recently, staff felt more competent to provide care for service users. In the HIC European countries where mhGAP was not being rolled out, PCPs reported that limited training in mental health was provided by different state or specialized institutions once their basic professional training had been completed, and that there were generally few to no incentives, nor obligations to attend:

“*I attended some courses on mental health themes within the structured training required for the specialization*. *Recently*, *I have not attended any training course on these themes*. *To my knowledge*, *there are no organized trainings available for general practitioners on mental health issues*. *We are forced to educate ourselves independently*,”

(*PCP*, *man*, *Italy)*.

Information was not able for the Italian setting regarding whether such trainings were available, but the PCP was not aware of them, or organized trainings were not delivered as the PCP stated.

PCPs expressed a strong desire for more training, especially practical training, such as on how to communicate with service users or on how to deal with challenging situations. PCPs also desired more supervision by specialist services when providing care for service users. Overall, PCPs from all sites complained of a lack of communication and cooperation with specialized mental health services.

*Service provider wellbeing and burnout*. With the exception of the Czech Republic, PCPs from all other sites reported exhaustion and burnout among staff due to workload, constrained time for assessment, lack of personnel, lack of organizational support, and criticism from patients. “*Burnout leads to less patience and less concentration on work*. *The quality of services gets affected*,” *(PCP*, *man*, *India)*. *Similarly*, “*when we are stressed*, *attention towards patient will be less as we have intention to finish our works*,” *(Pharmacist*, *woman*, *India)*. None of the countries reported having structured guidelines to address staff burnout. However, in Hungary, India and Tunisia, staff were trying to protect their wellbeing through ensuring support and having regular discussions about their difficulties. In India and Nepal, some structured trainings had been organized for PCPs to improve their psychological wellbeing, but these were only one-off events and not sustained.

### Perspectives of people with mental illness and their families

#### Experiences of stigma and discrimination in primary care settings

When asked about their experiences of treatment at their primary care center, service users reported a range of different experiences. Notably, most of the experiences reported were positive. Across all sites, several service users said that PCPs were ‘nice’ and ‘friendly’, or they specifically denied being treated in a discriminatory way. As one woman diagnosed with somatoform disorder from India put it: “*Generally*, *the PCPs treat us the same as other patients*. *Why should they treat us differently*?” A small number of service users in Tunisia and Lebanon emphasized that PCPs were *more* understanding and accepting than other health staff (including mental health specialists): “*They treat me like a human being*, *unlike other doctors*,” *(Service user*, *woman*, *Lebanon)*.

Service users in Tunisia, Lebanon, India, Nepal, and Hungary shared limited direct experiences of stigmatizing or discriminatory behavior from PCPs. Family members who were interviewed in Tunisia, India, Nepal and Hungary also reported no discriminatory behavior by PCPs. However, stigma and discrimination experiences were reported by service users in the other settings. In the Czech Republic, some service users reported experiences such as physical problems being overlooked (possibly due to their mental health diagnosis), being advised to get an abortion, being discouraged from getting therapy because therapists are “*charlatans who don’t know what they do*”, receiving inadequate care, having diagnostic statements communicated to their employers against their will, and being treated with lack of empathy. One woman reported: “*my general practitioner told me ‘Get up’ and she called me a ‘weeper’ because she always made me cry when I came in*,” *(Service user*, *woman*, *Czech Republic)*. In Italy, some service users also reported lack of empathy by PCPs and certain experiences, such as post-partum depression, being minimized or dismissed. In Italy, a husband reported feeling blamed for his wife’s illness by their PCP. In Tunisia, there were no accounts of discriminatory behavior by PCPs, but some participants reported anticipated discrimination or fears that other people in the waiting room may come to know they had a mental disorder. One woman with a common mental disorder explained: “*Sometimes*, *when I come to sign up or something*, *or to take my medicine*, *I don’t want them to see that it’s medication for my nerves and all*… *(…) I don’t want anybody to know me … they would say she’s …*. *sick… she has a problem with her nerves*,” *(Service user*, *woman*, *Tunisia)*. Similarly, another PCP responded, “*Other patients sitting in the waiting room make remarks on people with mental disorders and in many occasions*, *they refuse the suggested treatment because of it*,” *(PCP*, *man*, *Hungary)*.

Participants from two sites (Tunisia and Czech Republic) compared the discrimination experienced in primary care centers with their experiences in specialty psychiatric services. They reported mental health specialists’ behavior that was perceived as devaluing and discriminatory by service users. This included experiences such as being ignored, belittled, or yelled at and treated ‘violently’ in the context of involuntary treatment. No detailed information on this subject was available from the other sites.

#### Expectations for mental health services in primary care

Service users did not report expectations that mental healthcare would be offered by PCPs. Instead, mental healthcare provision was primarily associated with specialized services. In Tunisia and the Czech Republic, service users thought that while PCPs can offer help and encouragement, they are eventually unable to understand mental health problems to the same extent as psychiatrists. A woman with a common mental illness from Tunisia stated, “*only a psychiatrist can understand psychological problems*, *not GPs*,” *(Service user*, *woman*, *Tunisia)*. Similarly, “*I don’t go to the GP*, *I go straight to the specialist because I don’t trust her* [the PCP]…,” *(Service user*, *man*, *Czech Republic)*.

#### Experiences of stigma and discrimination outside healthcare

Service users reported negative experiences in interaction with family members, neighbors, people at work, or the general public, making this a very salient and cross-cutting aspect of the stigma experience. Reports by service users were corroborated by their family members. Negative reactions from family members appeared to be particularly salient and burdensome for service users. In Tunisia, for example, participants described not being taken seriously, having all their actions be attributed to them taking medication or being ill, or family members not accepting their illness. Several also experienced blame or critical comments, were excluded from family gatherings, or were being called derogatory terms. For example, one woman from Tunisia with severe mental illness said:

“*(My sister) would avoid me … every time I talk*, *she would criticize me … she would always tell me that I’m not clean or that I’m not taking care of myself and that my house is dirty … (once) it was dinner time*, *I was sitting on the dinner table …*, *my older sister looked at me and asked me to leave because they are going to have dinner … I looked at her*, *teared up and stood up … then I went home*,”

*(Service user*, *woman*, *Tunisia)*.

“*Even my best friend*, *said ‘you’re crazy*, *you go to (name of psychiatric hospital)*, *your siblings are crazy*, *your whole family is crazy*. *Who would marry into your family*? *Who would marry you*? *If you’re a crazy person who seeks treatment at (name if psychiatric hospital)*, *who would marry you*? *And your siblings are crazy*.*’ She said that to me*. *And since she said that*, *I broke down… when she said that word*, *I mean*, *I mean*, *… Glory to God*, *I mean*, *I broke down to pieces*.

*(Service user*, *woman*, *Tunisia)*

A family caregiver described how her brother, who has mental illness, was being stigmatized by his own family members. “*I noticed something from the family when he visits them*, *for example … they immediately call me and tell me ‘Your brother came here*, *he came and he stayed*… *It’s like a monster visited them … You know*? *(Caregiver*, *woman*, *Tunisia)*. Similarly, another family caregiver said, “*People say that I am a sister of a drunkard*. *They call us the family of a drunkard*.*’” (Family member*, *women*, *Nepal)*.

In Hungary and the Czech Republic as well, service users described being blamed, judged or avoided by some of their family members. In India, some service users reported that their family members did not take care of them well. Some of them also faced physical violence, were scolded or not treated with respect. Across sites, service users further reported negative reactions from friends, neighbors, colleagues at work or the general public. They spoke about being avoided and shunned, being mocked and being called names.

In Nepal, service users were concerned that others might find out about their illness, as this would lead to problems in employment. Discrimination, such as name calling, avoidance, or lack of understanding at the workplace was reported from Hungary, Italy and the Czech Republic. Service users’ accounts of stigma and discrimination were largely confirmed by their family members. In addition, family members of service users were themselves affected by stigma. For example, family members in Nepal reported being called names, receiving blame, losing a job or being rejected from public festivals. In Nepal, this was mostly in the case of family members of people with serious mental illness.

Some service users, on the other hand, shared positive experiences and said they were treated well by family members, friends and neighbors.

#### Anticipated discrimination and emotional consequences of stigma

Experiences of anticipated discrimination were reported in Tunisia, Nepal, India, Lebanon, and Italy. Particularly salient were fears of other people finding out about the illness and anticipated discrimination, i.e., fears of labelling, being seen as different, or being excluded. This also applied to marriages. One Syrian refugee with a common mental disorder said that she concealed her mental health problems from her husband for fear of being abandoned: "*My husband doesn’t know about this problem; I don’t show him (…) because I am afraid that he might leave me for this*,” *(Service user*, *woman*, *Lebanon)*. Service users in Tunisia also reported fears of being labelled ‘crazy’ and having reduced marital prospects through attending mental health services. Similar problems were also reported in Nepal.

In India, service users were scared of how they would be treated in society and would therefore withdraw socially, not attending functions or events. Some of them preferred not to go outside their home. Some interviewed service users expressed that they felt helpless or tense when others treated them differently and tried to avoid social situations. In Nepal, one woman with a common mental illness explained, “*I always remain alone*. *Others treat me as a different person*. *So*, *I won’t go to interact with them*.” Other service users in Nepal voiced anticipated discrimination in thoughts such as, “*I might be seen as crazy*”, “*I might be seen as weak for having mental health problems*,” and “*It might harm my chances when applying for jobs*,” *(Service user*, *man*, *Nepal)*. To prevent disclosure, service users would often stop their medication or stop treatment altogether. Service users also felt disempowered in the health system, feeling unable to make their own decisions. People with severe mental disorders, in particular, reported severely constrained social lives.

Service users in Tunisia reported fears of being labelled as mentally ill in the waiting room of primary care centers; they expressed their apprehension that they might be seen by neighbors or friends while getting psychiatric medication in primary care centers. Tunisian service users spoke about how this anticipated stigma affected them emotionally and impacted their self-esteem. For example, they would cry when faced with harassment, feel out-of-place, keep to themselves, or become more self-critical. For example, one woman from Tunisia with a common mental disorder said “*sometimes I cry*, *I feel bad about myself…I cry … I feel like I’m missing something (…) I would cry and then I would take it all out on myself*,” *(Service user*, *woman*, *Tunisia)*.

Service users’ experiences of anticipated stigma were mirrored by family members where such information was available. In Nepal, family members also tried to hide the illness as much as possible due to negative societal attitudes towards service users. They were reluctant to tell neighbors and community members. Family members in Tunisia confirmed the fears of disclosure they witnessed in their ill family members.

### Stakeholder perspectives on “what needs to be done”

#### Primary care providers’ perspectives

All healthcare staff from frontline workers to managers called for more training of PCPs in mental healthcare. In the Czech Republic, one primary care manager suggested that, “*there should be mental health training for GPs*. *If the GP was more educated in psychiatry*, *he could solve many more problems (*…*)*,” *(Manager*, *gender not recorded*, *Czech Republic)*. In Tunisia, India and Lebanon, PCPs also emphasized the need for regular and continuous training and supervision for PCPs on topics such as psychiatric medication, providing psychosocial support to someone in need, communicating with service users, and addressing stigma and self-care. PCPs also called for more collaboration with and supervision by mental health specialists:

“*The main reason is the lack of collaboration between general practitioners and MH professionals*. *Collaboration with mental health services would be relevant for the diagnosis*, *management and psychological support of patients with mental illnesses*. *Referrals in psychiatry are difficult*, *waiting times are too long*, *telephone consultations are rare*,”

*(PCP*, *woman*, *Italy)*.

“*I am sure that all doctors and medical staff need to have a [mental health professional] mentor in the second and third line of healthcare*, *so that they do not feel alone*, *so that they can improve the contact or communication between the various lines*,”

*(Program manager*, *man*, *Tunisia)*.

“*Yes*, *we don’t have a specialist present full time*. *We have received training in mhGAP but this is not our profession*. *When we have a specialized person like a psychologist in the center to whom we can refer*, *the work would be better*,”

*(PCP*, *woman*, *Lebanon)*.

A nurse in Lebanon explained that she considered continuous evaluation of their work as essential as the training, and that more training on stigma reduction and how to use self-care skills at work to avoid burnout were required. In Nepal, one PCP reported greater enthusiasm for providing mental healthcare after hearing recovery testimonials from service users:

“*When seeing those people who have recovered*, *we got the proof that their condition can improve if they get timely treatment and timely counseling … We got to know how the patients feel and what drives them to do certain things*, *what triggers depression*. *We got to interact with patients who previously had postpartum depression and postpartum psychosis … I felt really bad to know about the challenges they face in society*. *I could empathize with them and realize how they might have felt*. *So*, *I felt happy to be able to provide service to people with such problems*,”

*(PCP*, *man*, *Nepal)*.

In terms of structural changes for the work in PCPs, one general practitioner in Italy suggested providing suitable environmental support such as having a separate room for counselling to ensure privacy. Some PCPs in HICs suggested changes to the way health services were organized to include direct telephone consultations with psychiatrists or mental health specialists to reduce referrals and seek advice, or having outpatient services offered by mental healthcare specialists in PCPs. Some PCPs in Italy also suggested developing scales and tools to help them diagnose mental illness.

Additional findings included calls for psychoeducation for service users and their families, using creative techniques such as movies or videos on mental health to raise awareness (Lebanon), and public education campaigns through media such as television and radio. PCPs in India and Nepal further suggested incorporating mental health education in school curricula to address stigma and providing mental health interventions for children in schools.

#### Perspectives of people with mental illness and their family members

When asked about what would help to address the problems identified, service users from India and Nepal emphasized that their communities needed to receive better information about mental illness and associated treatments in order to increase acceptance of mental illness and utilization of healthcare services. Service users in Nepal suggested that there should be training programs in villages, so that people can understand that taking medication for mental illness can heal, that it does not make one ‘crazy,’ and that people should not feel scared of someone finding out what the medicine was actually for. Similarly, service users in India highlighted that there was a need for improving understanding of the problems of service users among community members and those affected while fostering the use of available treatments to reduce false beliefs. For example, one caregiver from India stated: “*If we get information on how to take care of mental illness*, *it will be really helpful*. *So*, *training should be given for spreading health information among people*,” *(Caregiver*, *man*, *India)*. On a similar note, service users in the Czech Republic suggested that bringing about health system-level changes to address low knowledge about mental illness and community services would help to address stigma.

Other suggestions, particularly from Italy, Hungary and Tunisia, concerned training for PCPs in order to improve their effective communication and empathy in interactions with service users. In Italy, service users spoke about the importance of trust and adequate time in interactions, that PCPs should listen to their patients more, and that PCPs should not scare people when talking about mental illness. An Italian man who was a caregiver for a service users requested that primary care physicians should be able to refer family members to additional support or learning resources on how to behave with someone with mental health problems. In Tunisia, a service user suggested that it would help if doctors asked about the wellbeing of the service users: “*Of course*, *that he would ask me about how I feel or if I got better or if I’m working or if I do this or that … But I don’t get those questions*,” *(Service users*, *man*, *Tunisia)*.

Evidence from implemented stigma-reduction interventions in the Nepal study site further suggested that service users felt that knowing someone who had recovered from a mental illness or hearing success stories from health workers were helpful in terms of supporting service users with their experiences of stigma. They felt more comfortable knowing that others had recovered with treatment, that they were now participating in training, and that they were even working in the health sector. Service users also mentioned counselling and family support as having helped their recovery.

## Discussion

This study set out to generate evidence on how to improve primary-care based mental health services by reducing stigma in primary care settings. Based on qualitative data collected from some primary care providers and service users in seven countries in Africa, Asia, and Europe with varying levels of mental health integration in primary care, we identified several stigma-related barriers to optimal care that could be targeted within interventions. Across all countries, PCPs stated their motivation to support service users and treat them fairly. However, our analysis also revealed stereotypes and potentially stigmatizing attitudes and behaviors among PCPs that may have impeded the quality of care provided. For example, findings suggest that stereotypes such as “mentally ill people are dangerous” or “mental illness is incurable” and fears about interacting with service users increased some PCPs’ hesitation to provide care to service users. These findings are in line with prior research on attitudes of primary care workers towards service users in these settings and more broadly among PCPs [4, 9, 33, 34]. Although a study in the Czech Republic reported that stigmatizing attitudes were less severe among medical doctors—despite still being quite high—compared to the non-medical public, we do not have public attitudes to comment on whether the expressed attitudes among PCPs differ from the general public [31].

In a context of poor resources, service users were also perceived as burdensome, time consuming, or annoying by some PCPs, particularly in LMIC settings. PCPs across most sites acknowledged that their own wellbeing was affected due to high work pressures and that this had a negative impact on the care they provided. Ultimately, most PCPs did not see it as their role to provide mental healthcare in primary care centers or felt inadequately trained or supported to do so. In Nepal and Lebanon, where mhGAP training had been carried out, PCPs reported an improved ability to provide care for service users, reduce stigma and therefore improve mental healthcare offered at PCPs. Moreover, in Nepal, mhGAP trainings were paired with social contact stigma reduction interventions [15, 22].

Looking at experiences of service users and family members, it was striking that there were few experiences of discrimination reported from primary care centers. On the contrary, most service users said they were happy with the way they were treated there, even though there were some complaints about systemic issues such as waiting times or availability of medication. More clear-cut examples of discriminatory behavior in primary care centers were reported from the Czech Republic and Italy. In Tunisia, some service users reported fear of disclosure and anticipated discrimination through their primary care treatment, such as worries about being identified and labelled as mentally ill in the waiting room. This might have been compounded by structural issues such as patient files visibly identifying service users in primary care settings in Tunisia.

Among service users, the most common forms of stigma and discrimination described were enacted outside the health services by family members, friends, neighbors, or the general public. Also, internalized stigma and anticipated discrimination were cross-cutting among the sites. This had powerful consequences such as reducing emotional wellbeing and self-esteem, eventually leading to social withdrawal and reduced help-seeking. Overall, there was commonality in the way stigma manifested. The types of discriminatory experiences reported by service users and family members outside healthcare were also quite similar; e.g., being called names or being avoided were particularly salient. Stereotypes, attitudes, and behaviors among PCPs were also shared, such as beliefs that “mentally ill people are dangerous” and fears of interactions with service users. Some ideas, such as “mental illness is not curable,” were limited to South Asian sites (India and Nepal) and Tunisia.

Taken together, these findings suggest that: (i) there is enough similarity in the problems identified across sites to justify multi-country anti-stigma interventions, and (ii) that it is very important to also identify local factors influencing stigma and adapt interventions accordingly. Future studies should examine the cultural and contextual factors contributing to stigma in primary care with larger study samples in order to develop specific guidelines for cultural adaptations [6, 35].

### Limitations of the study

Our study findings are limited by heterogeneity in the employed study methodology across sites. As the study was carried out without external funding in most sites, participant numbers varied depending on available site resources. Despite efforts to harmonize study methodology by providing questionnaire guidelines, data analysis tools and methodological support, there were still cross-site variations in terms of research experience of interviewers, depth of probing on certain topics, and other aspects of research implementation. The samples interviewed in some sites were small, and it is possible that more experiences of discrimination would have been identified in a larger sample of service users. Due to the small sample sizes in some sites, differing levels of detail in the data and the wide range of aspects of stigma studied, there are likely other commonalities and differences that could be unveiled through more in-depth work in each site.

Although the diversity of settings covering low-income, lower-middle-income, upper-middle-income, and high-income was a strength, the diversity of settings also meant that there were differences in the backgrounds of PCPs and differences in health system structures across settings. There was also heterogeneity in relation to the mental health background of the PCPs. This would suggest that those with mental health training have more information and skills in the treatment of people living with mental illness, which can influence their attitude towards patients, potentially decreasing stigmatization. We did not adequately sample PCPs with a background in mental healthcare vs. those without a mental health background in order to reach meaning saturation of these subgroups across all sites. For example, in Hungary, all four PCPs have professional contact with the community psychiatry center of the district, which shows a more open, tolerant attitude towards patients living with mental illness, and this is not likely to be representative of PCPs across the country. There was also differential understanding of topics across sites. For example, in India, although they were aware of social stigma, there was less understanding of the concept of structural stigma.

In Lebanon, the primary care doctors were general practitioners who had received mhGAP trainings. However, general practitioners throughout the country had not universally been exposed to mhGAP training at the time of the study, therefore results cannot be generalized to PCPs without any mental health training. In Nepal, the secondary data were extracted from multiple interviews that was conducted with health workers, patients receiving mental health treatment in primary healthcare facilities, and service users and family members involved in anti-stigma interventions in Nepal.

A limitation of the Lebanon study was that qualitative interviews were limited to two urban areas with higher economic status than many other areas of the country. In the future, underprivileged areas should be investigated further and may reveal differences in the findings reported here. Similarly, in India, because the study site has more than 60 primary care centers, further studies can be conducted by including more primary care centers of the district on a larger scale, and similar studies can also be conducted in other districts of the state to enhance generalizability.

Similar to challenges with the heterogeneity within and between sites related to care providers, the service users and their family members could not capture the diversity of demographic differences within and between countries. In India, the interviewed service users and family members were from a rural background. As a result of this, we found that the service users and their family members had low levels of mental health literacy and limited knowledge of their own conditions and how treatment and attitudes may have been influenced by their diagnoses. The service users for the study were identified by psychiatric diagnoses on mental health outpatient cards. However, information on the diagnoses was often incomplete and the service users were rarely aware of the exact diagnosis for their conditions. In Nepal, as the PRIME intervention focused on 4 priority disorders (depression, alcohol use disorder, psychoses, and epilepsy), all the patient and service user participants were representative of only these disorders. In addition, all the participants interviewed were above 18 years of age and so represent only the adult population. There are a number of limitations that should be considered when interpreting results, such as the service users reporting lack of stigmatizing experiences. Because most interviews were conducted by clinician-researchers at primary care centers, desirability bias could have masked stigmatizing attitudes among PCPs as well as stigma experiences among service users and their family members. For example, in Lebanon, contact with PCPs was established through supervisors, which may bias responses to more positive attitudes or greater willingness to take on mental health services than participants may have reported if not referred by their supervisors. In India, the context under which the interviews were conducted likely influenced the level of detail and type of responses. Because of high clinical workloads and shortage of time for attending interviews, the qualitative interviews conducted in clinical sites were abbreviated.

Moreover, because in most sites service users were nominated by staff at the primary care centers or known to the study teams, they may reflect a biased sample who have more positive experiences of mental health services in primary care. For example, in Lebanon, service users were reached by contacting primary care facility focal points who then referred the service users for the qualitative interviews. This recruitment technique may have reduced the likelihood of providing comments that were critical of services received at those clinics. Another consideration is that by conducting the interviews in primary care centers in some sites as well as potentially associating interviewers with primary care staff, the service users may have been concerned that negative responses would impact their care. In the consent forms, they were told their care would not be impacted, but the context of the interviews may have not been reassuring for this point.

Similarly, the level of motivation of primary care centers to train more in mental health may be biased based on the sites participating. In some sites, the primary care centers taking part in this study had purposely been chosen as pilot sites for an intervention to reduce stigma based on their expressed interest and reliability and may therefore not have been representative of other primary care centers in their site. Another factor to consider is gender. The vast majority of PCPs interviewed (75%) were women. We do not know if men in these roles would have had similar perspectives.

Ultimately, the study provides an overview of some the types and diversity of attitudes and experiences related to stigma in primary care settings. However, given the type of data collection and methods described here, the findings should not be viewed as comprehensive and universal throughout the countries studied.

### Recommendations

This study provides a number of lessons for both researchers and for practitioners interested in reducing mental illness stigma. We summarize these key recommendations below.

### Research recommendations

1. For qualitative research on attitudes and experiences of service users, clinician-researchers may not be the ideal sole qualitative interviewers because of power differentials and expectations of service users about how to respond to persons they perceive as part of the healthcare system. For future research, it would be helpful to have qualitative researchers who are not part of the health system be included as interviewers. These interviews could also be conducted in non-clinical settings to help reduce the perception that the research team is directly linked to clinical care providers. Although the consent information in our study indicated that responses would be kept confidential, this may not have been the perception when interviews were conducted in clinical settings and sometimes by researchers who also worked in those facilities.
2. Collaboration with service users for qualitative data collection may contribute to reduced bias in responses of service users. Research teams could consider recruiting and training service users as qualitative researchers. Some of the sites in the study trained clinicians without prior qualitative experience to conduct interviews for this study. Those training efforts could be directed to service users. Similarly, service users could have been involved in the qualitative analysis to reflect upon whether the responses of participants were consistent with local experiences or if there may have been bias introduced by the recruitment and data collection strategies.

### Stigma reduction and mental healthcare strengthening recommendations

1. Equip PCPs for taking on basic mental healthcare as suggested by the WHO [13] by providing training on identification of mental disorders, basic mental health treatments, psychosocial counselling, and communication skills as outlined in mhGAP training. For example, the statements by PCPs in Nepal and Lebanon, where mhGAP training had already been implemented, highlight that they felt much more confident and equipped to take on a role in providing mental healthcare in their site after their training—a finding which has also been documented in respective evaluation studies [36]. This suggests that implementing mhGAP training would seem to be a valuable next step in providing basic mental health training to PCPs in primary care settings where this has not yet been done. These mental health trainings could also be brought into pre-service training for health workers.
2. Prepare PCPs for being able to handle a wider range of interactions with service users with mental illness (according to local need) with empathy, skill, and confidence, including managing crisis situations. In a study of PCPs trained in mental healthcare in Liberia, Uganda, and Nepal, lower stigmatizing attitudes were associated with greater common factors of mental healthcare, such as empathy, collaboration, and promoting hope for recovery [37]. The importance of training PCPs in understanding and empathetic communication was mentioned specifically by several service users. New initiatives such as the WHO Ensuring Quality in Psychological Support (EQUIP) platform could be used to enhance empathy and communication skills [38].
3. Address universal and locally specific stereotypes, attitudinal barriers and stigmatizing behaviors among PCPs [6]. Our findings highlight the importance of addressing universally held stereotypes such as service users being dangerous or mental illness not being treatable, but also identifying any locally specific myths and addressing what matters most to service users and PCPs in different settings [39]. Based on the existing evidence in anti-stigma interventions in healthcare and particularly the findings of previous anti-stigma work done in one of our study sites, Nepal, we further suggest addressing stigmatizing stereotypes, attitudes and behaviors among PCPs through contact-based interventions employing trained service users [15, 22].
4. Train PCPs to support service users and families with the significant experiences of stigma enacted outside healthcare by providing psychoeducation, providing practical guidance and addressing internalized stigma [40]. To address internalized stigma, interventions should also cover basic counselling skills and simple interventions for self-esteem building and empowerment. One of the particularly salient findings across sites was experience of stigma enacted by family members, who were often carrying a large burden of care without adequate information or support. Training interventions for PCPs should therefore comprise delivering psychoeducation to service users and family members who often accompany service users to appointments and guidance with handling challenging situations at home. Findings from Nepal suggest that training service users for social contact interventions with PCPs should also include family members, which has the potential to also reduce family-based discrimination [19]. Similar interventions have been suggested and tried in the training of community mental health workers, e.g., in India [12] and in the International Federation of Anti-Leprosy Associations’ (ILEP) stigma reduction guidelines for health workers [41].
5. Assure that PCPs have ongoing support. Ongoing supervision and support are important for appropriate quality of care and to monitor motivation, treatment quality, and potential burnout as PCPs become more engaged in mental health services. There are many different models for this that could be adapted to the different countries; the issue is not just about training, but ongoing support for PCPs [42, 43].
6. In addition to training interventions targeted at individual health workers, interventions at the structural level are required. Primary care managers should review the work processes in their health facilities to identify any procedures that might expose service users to stigma, such as visibly marked patient files increasing fears of disclosure or consultation rooms lacking privacy. Checklists and tools should be developed or adapted to capture these aspects of structural stigma. Structural interventions should also seek to address PCP burnout and wellbeing by aiming to keep workloads manageable, creating support structures for staff, and teaching skills on self-care and burnout prevention.

## Conclusions

Our findings suggest that although the stigma enacted by primary care providers was modest and service users were largely happy with the treatment that they were receiving at their primary care centers, stigma still represents a prominent issue in the lives of service users with mental illness. This was demonstrated by the high levels of discrimination reported from families and community members, as well as considerable internalized and anticipated stigma across sites. It would therefore be wrong to conclude that there is no need for interventions in primary care to address stigma. On the contrary, our findings suggest that training interventions in primary care are necessary and very much requested by primary care providers. However, rather than just focusing on reducing stereotypes and improving attitudes alone, skill development and structural changes are needed to assure quality, supportive, and effective mental health services in primary care settings.

## Supporting information

S1 Table Consolidated criteria for reporting qualitative studies (COREQ): 32-item checklist. (DOCX)
