# Supplementary material for: Perspectives of healthcare providers, service users, and family members about mental illness stigma in primary care settings: A multi-site qualitative study of seven countries in Africa, Asia, and Europe
Source: PLoS One. 2021 Oct 27;16(10):e0258729. doi: 10.1371/journal.pone.0258729 (PMC8550394; doi:10.1371/journal.pone.0258729)
Supplement: S1 Table — (DOCX) [file pone.0258729.s001.docx]

**Consolidated criteria for reporting qualitative studies (COREQ): 32-item checklist**

Developed from:

Tong A, Sainsbury P, Craig J. Consolidated criteria for reporting qualitative research (COREQ): a 32-item checklist for interviews and focus groups. *International Journal for Quality in Health Care*. 2007. Volume 19, Number 6: pp. 349 – 357

| **No.  Item** | **Criteria description** | Study Information |
| --- | --- | --- |
| **Domain 1: Research team and reﬂexivity** |  |  |
| *Personal Characteristics* |  |  |
| 1. Interviewer/ facilitator | Which author/s conducted the interview or focus group? | Czech Republic: Researchers with a master degree or higher  Hungary: One senior psychiatrist, one clinical psychologist-trainee  India: Two Junior research fellows; Two Junior research fellows with Masters and MPhil. level training supervised project lead (Associate professor).  Italy: a psychiatrist in training and a consultant psychiatrist  Lebanon: Research assistants with bachelor’s and Master’s level of education  Nepal: Research assistants with Bachelor’s and Master’s level of education  Tunisia: Senior psychiatrists |
| 2. Credentials | What were the researcher’s credentials? E.g. PhD, MD | Czech Republic: Master or higher (PhD)  Hungary: MD, psychologist MA  India: Masters, M.Phil., PhD  Italy: MD, MD PhD  Lebanon : MD, PhD, MSC, MPH  Nepal: BSW, BPH, MA, MSC  Tunisia: MD |
| 3. Occupation | What was their occupation at the time of the study? | Czech Republic: Researchers at the National Institute of Mental Health, the Czech Republic  Hungary: Psychiatrist and clinical psychologist-trainee at Awakenings Foundation (Semmelweis University Community Psychiatric Centre)  India: Two Junior Research Fellow in PRIMARY project led by Associate professor, Department. of. Psychiatric Social Work, NIMHANS.  Italy: psychiatrist in training at the Psychiatric Specialization School, University of Verona; Associate Professor of Psychiatry, University of Verona  Lebanon: Research assistant and Service Development coordinators and Public Health Officers  Nepal: Research assistant and research officers for a mental health NGO  Tunisia: Clinician Researcher at Razi Hospital La Manouba |
| 4. Gender | Was the researcher male or female? | Czech Republic: Male and female researchers  Hungary: Female researchers  India: Male and female researchers  Italy: Male researchers  Lebanon: Male and female researchers  Nepal: Male and female researchers  Tunisia: Female researchers |
| 5. Experience and training | What experience or training did the researcher have? | Czech Republic: Personal experience in qualitative research, some have therapeutical training  Hungary: Personal experience, but no special training in qualitative research, training in psychotherapy  India: 2 years research experience. In addition,10 days of special training on qualitative research and the whole process was supervised by the research leads who have expertise in qualitative research.  Italy: A long-term experience in quantitative research in mental health, but little research in qualitative studies; no previous research experience  Lebanon: 3 years research experience  Nepal: Approximately 2 weeks of qualitative research training  Tunisia: Qualitative research training, has led interviews and focus groups for previous research |
| *Relationship with participants* |  |  |
| 6. Relationship established | Was a relationship established prior to study commencement? | Czech Republic: Only in a minority of participants included in a study  Hungary: yes, with all the service users and the PC staff a relationship was established before the study begun  India: Firstly, the associate mental health professionals of the study sites was approached and in coordination with them, relationship was established with medical officers of each PHC for the study  Italy: yes, with service users and caregivers involved in the research a relationship was established before the study  Lebanon: Previous collaborations between the NMHP team and PHC service providers. No prior relationship with service users and caregivers.  Nepal: With project staffs and trainers who we involved in a prior research project  Tunisia: only to the lead primary care clinician, the associated mental health professional, and the program manager, not to the rest of the study participants |
| 7. Participant knowledge of the interviewer | What did the participants know about the researcher? e.g., personal goals, reasons for doing the research | Czech Republic: no knowledge except for researcher’s affiliation  Hungary: they did not have knowledge about personal goals, but the goals of the research (assess stigma) were transparent  India: The associate mental health professionals’ team at the site briefed the primary health care medical officers about the study purpose and introduced the interviewer to the primary health care team. Permission was sought from the state health ministry before the field visit.  Italy: participants had no knowledge on researchers personal goals, but were informed on the goals of the research  Lebanon: The participants knew that the researchers were part of the ministry of public health working at the National Mental Health Programme and the reason is to understand Stigma at PHC.  Nepal: The participants knew that the researchers were staff of a local non-governmental organization that coordinating and funding mental health services in the district.  Tunisia: they did not have knowledge about personal goals, |
| 8. Interviewer characteristics | What characteristics were reported about the interviewer/facilitator? e.g., Bias, assumptions, reasons and interests in the research topic | Czech Republic: interest in stigma research; one of the researchers has been personally involved also as a vocational therapist  Hungary: interest of the stigma connected to people living with mental health disorders  India: Each Interview took on an average 60-90 minutes time and researcher managed to conduct interviews with sufficient breaks to minimize distractions and bias. Site leads had keen interest on the primary health care staff perspective towards quality of mental health care availability at local, as lead has more than 15 years of work experience in managing the psychosocial aspects people with mental health problem who visits tertiary care.  Italy: Interest in mental health service research and research on mental illness stigma and discrimination  Lebanon: Interest in mental health service research and in line with the objectives of the National Strategy for mental health  Nepal: Interest in mental health and stigma, and service user rights  Tunisia: long standing interest in the research topic based on the work in the mental health sector and being witness of the stigma attached to mental illness |
| **Domain 2: study design** |  |  |
| *Theoretical framework* |  |  |
| 9. Methodological orientation and Theory | What methodological orientation was stated to underpin the study? e.g. grounded theory, discourse analysis, ethnography, phenomenology, content analysis | The study used a combination of deductive and inductive methodology in a framework analysis approach. That is, a basic thematic coding framework was developed together with the sites based on field experience in the sites and existing research evidence, for example detailed inductive qualitative work on stigma among people with mental illness in India (Koschorke et al. 2014, <https://doi.org/10.1016/j.socscimed.2014.10.035>). In addition, all sites carried out inductive coding of the data to validate/adapt the coding scheme for the local context and identify additional codes. In this way, the methodological approach sought to ensure that data could be pooled and compared across diverse sites as well as allowing for local and cultural variation. |
| *Participant selection* |  |  |
| 10. Sampling | How were participants selected? e.g. purposive, convenience, consecutive, snowball | Czech Republic: combination of convenience and purposive among healthcare professionals  Hungary: Convenience  India: Purposive sampling  Italy: Convenience  Lebanon: Convenience  Nepal: Purposive, convenience  Tunisia: convenience |
| 11. Method of approach | How were participants approached? e.g. face-to-face, telephone, mail, email | Czech Republic: Face to face  Hungary: face to face  India: Face to face  Italy: face-to-face and telephone  Lebanon: face-to-face and telephone  Nepal: face to face  Tunisia: face to face |
| 12. Sample size | How many participants were in the study? | See Table 2 in the manuscript |
| 13. Non-participation | How many people refused to participate or dropped out? Reasons? | Czech Republic: None  Hungary: None  India: 2 service users and 1 service provider (reasons not recorded)  Italy: None  Lebanon: Some service users refused to participate because recruitment was done by primary care staff and they reported feeling uncomfortable speaking on topics of stigma with the primary care staff. The number of refusals was not recorded.  Nepal: None  Tunisia: 2 Service providers: time constraints, not interested; 2 service users: not interested |
| *Setting* |  |  |
| 14. Setting of data collection | Where was the data collected? e.g., home, clinic, workplace | Czech Republic: clinic (service users), workplace (primary care workers)  Hungary: health care professionals: ambulance of GP, service users: clinic, at home  India: Data from Service provider, Service users and family members was collected at primary health care centers  Italy: clinic and workplace  Lebanon: primary care center  Nepal: health facilities, community spaces, home of some participants  Tunisia: for service providers at their workplace, for service users at the primary care center |
| 15. Presence of non-participants | Was anyone else present besides the participants and researchers? | Czech Republic: no  Hungary: no  India: no  Italy: no  Lebanon: no  Nepal: in case of service users, sometimes caregivers were present  Tunisia: no |
| 16. Description of sample | What are the important characteristics of the sample? e.g., demographic data, date | Demographic data are included in Table 3. Data collection dates are provided below.  Czech Republic: July-November 2017  Hungary: October-December 2017  India: June 2018 to January 2019  Italy: October 2018 to March 2019  Lebanon: October to December 2018  Nepal: 2014; February 2016 to August 2018  Tunisia: March to September 2018 |
| *Data collection* |  |  |
| 17. Interview guide | Were questions, prompts, guides provided by the authors? Was it pilot tested? | Czech Republic: yes  Hungary: yes  India: yes, sample interviews were conducted before the actual interviews for pilot testing using the interview guide  Italy: yes  Lebanon: yes and translated to Arabic  Nepal: yes  Tunisia: yes |
| 18. Repeat interviews | Were repeat interviews carried out? If yes, how many? | Czech Republic: no  Hungary: no  India: no  Italy: no  Lebanon: no  Nepal: yes, 15 healthcare workers; 12 service users and their caregivers  Tunisia: no |
| 19. Audio/visual recording | Did the research use audio or visual recording to collect the data? | Czech Republic: audio recording  Hungary: audio recording (healthcare professionals only)  India: audio recording  Italy: none  Lebanon: audio recording  Nepal: audio recording  Tunisia: audio recording |
| 20. Field notes | Were ﬁeld notes made during and/or after the interview or focus group? | Czech Republic: no  Hungary: yes  India: yes  Italy: no  Lebanon: no  Nepal: yes  Tunisia: no |
| 21. Duration | What was the duration of the interviews or focus group? | Czech Republic: 30 - 60 minutes  Hungary: 45 - 90 minutes  India: 60 - 90 minutes  Italy: 30 - 60 minutes  Lebanon: 30 – 45 minutes  Nepal: 40 – 90 minutes  Tunisia: 30 – 120 minutes |
| 22. Data saturation | Was data saturation discussed? | As study samples in some sites were very small due to feasibility/resource constraints, it was not possible to aim for or examine data saturation at the site level or for minor themes. For the presence of major themes, such as experiences of stigma from community members (major headings in the results section), data saturation was achieved in the total/cross-country sample. |
| 23. Transcripts returned | Were transcripts returned to participants for comment and/or correction? | Czech Republic: no  Hungary: no  India: no, however the transcripts were reviewed independently by research team members.  Italy: no  Lebanon: no  Nepal: no  Tunisia: no |
| **Domain 3: analysis and ﬁndings** |  |  |
| *Data analysis* |  |  |
| 24. Number of data coders | How many data coders coded the data? | Six persons were involved in summarizing all data received from the sites. |
| 25. Description of the coding tree | Did authors provide a description of the coding tree? | A thematic coding framework was created jointly with the research teams in all sites to structure multiple-researcher coding. Inductive coding was used to identify additional codes relevant to the specific context in each site. The coding framework was charted into tables to compare various stakeholders’ experiences and views on key themes such as “attitudes of PCPs” or “experiences of stigma and discrimination in primary care”. In a second step, similarities and differences between research sites were mapped out on each theme. Relevant subthemes evident in the data were identified (e.g., different types of stereotypes evident in PCP narratives across countries). Cross-country findings on each theme were summarized for a) data from primary care providers (provider perspective) and b) interviews/focus groups with people with mental illness and family members (service user perspective) with a particular focus on findings that were salient across study sites and findings that appeared to be more locally specific. In a final step, implications for interventions to address stigma and discrimination in primary care were identified. |
| 26. Derivation of themes | Were themes identiﬁed in advance or derived from the data? | Both. All sites were provided with a pre-developed thematic coding framework and inductive coding was used to identify additional codes relevant to the specific context in each site. |
| 27. Software | What software, if applicable, was used to manage the data? | Each site used different software for their respective analyses. Results were then transferred into pre-developed data Excel summary sheets that were used for a cross-site analysis.. |
| 28. Participant checking | Did participants provide feedback on the ﬁndings? | Participants did not provide feedback on the findings. |
| *Reporting* |  |  |
| 29. Quotations presented | Were participant quotations presented to illustrate the themes/ﬁndings? Was each quotation identiﬁed? e.g. participant number | Quotations were presented to illustrate major themes and findings. Basic demographic description (gender, participant type, country) was given for the person making each of these statements. Participant numbers were used within sites but not pooled across sites for the cross-country write up. |
| 30. Data and ﬁndings consistent | Was there consistency between the data presented and the ﬁndings? | Yes |
| 31. Clarity of major themes | Were major themes clearly presented in the ﬁndings? | Yes (major headings in the results section) |
| 32. Clarity of minor themes | Is there a description of diverse cases or discussion of minor themes? | Yes (sub-headings in the results section) |
